# You Will Know That Despite Being HIV Positive You Are Not Alone: Qualitative Study to Inform Content of a Text Messaging Intervention to Improve Prevention of Mother-to-Child HIV Transmission

**DOI:** 10.2196/10671

**Published:** 2018-07-19

**Authors:** Jade Fairbanks, Kristin Beima-Sofie, Pamela Akinyi, Daniel Matemo, Jennifer A Unger, John Kinuthia, Gabrielle O'Malley, Alison L Drake, Grace John-Stewart, Keshet Ronen

**Affiliations:** ^1^ Department of Global Health University of Washington Seattle, WA United States; ^2^ Kenyatta National Hospital Nairobi Kenya; ^3^ Department of Obstetrics and Gynecology University of Washington Seattle, WA United States; ^4^ Department of Obstetrics and Gynecology University of Nairobi Nairobi Kenya; ^5^ Department of Epidemiology University of Washington Seattle, WA United States; ^6^ Department of Pediatrics University of Washington Seattle, WA United States; ^7^ Department of Medicine University of Washington Seattle, WA United States

**Keywords:** HIV, ART, PMTCT, SMS text messaging, adherence, retention

## Abstract

**Background:**

Prevention of mother-to-child HIV transmission (PMTCT) relies on long-term adherence to antiretroviral therapy (ART). Mobile health approaches, such as text messaging (short message service, SMS), may improve adherence in some clinical contexts, but it is unclear what SMS content is desired to improve PMTCT-ART adherence.

**Objective:**

We aimed to explore the SMS content preferences related to engagement in PMTCT care among women, male partners, and health care workers. The message content was used to inform an ongoing randomized trial to enhance the PMTCT-ART adherence.

**Methods:**

We conducted 10 focus group discussions with 87 HIV-infected pregnant or postpartum women and semistructured individual interviews with 15 male partners of HIV-infected women and 30 health care workers from HIV and maternal child health clinics in Kenya. All interviews were recorded, translated, and transcribed. We analyzed transcripts using deductive and inductive approaches to characterize women’s, partners’, and health care workers’ perceptions of text message content.

**Results:**

All women and male partners, and most health care workers viewed text messages as a useful strategy to improve engagement in PMTCT care. Women desired messages spanning 3 distinct content domains: (1) educational messages on PMTCT and maternal child health, (2) reminder messages regarding clinic visits and adherence, and (3) encouraging messages that provide emotional support. While all groups valued reminder and educational messages, women highlighted emotional support more than the other groups (partners or health care workers). In addition, women felt that encouraging messages would assist with acceptance of their HIV status, support disclosure, improve patient-provider relationship, and provide support for HIV-related challenges. All 3 groups valued not only messages to support PMTCT or HIV care but also messages that addressed general maternal child health topics, stressing that both HIV- and maternal child health–related messages should be part of an SMS system for PMTCT.

**Conclusions:**

Women, male partners, and health care workers endorsed SMS text messaging as a strategy to improve PMTCT and maternal child health outcomes. Our results highlight the specific ways in which text messaging can encourage and support HIV-infected women in PMTCT to remain in care, adhere to treatment, and care for themselves and their children.

**Trial Registration:**

ClinicalTrials.gov NCT02400671; https://clinicaltrials.gov/ct2/show/NCT02400671 (Archived by WebCite at http://www.webcitation.org/70W7SVIVJ)

## Introduction

Globally, over 90% of pediatric HIV infections are attributed to mother-to-child HIV transmission (MTCT), with 160,000 children newly infected with HIV in 2016 [1]. Lifelong antiretroviral therapy (ART; known as Option B+) is recommended for prevention of MTCT (PMTCT), but it depends on consistent retention in care and adherence to ART during pregnancy, postpartum, and beyond. Waning ART adherence and poor retention in care, particularly postpartum after the risk of MTCT diminishes, hinder the effectiveness of Option B+ [2-4]. Barriers to ART adherence in Option B+ stem from an interplay of sociocultural and structural factors [5-7]. Thus, identifying and addressing these barriers is important to improve PMTCT.

There is evidence from some randomized controlled trials (RCTs) and meta-analyses that short message service (SMS) text messages sent to individuals on ART may improve retention, adherence, and viral suppression in nonpregnant adults [8-10]. These findings, combined with the growing ubiquity and low cost of mobile phone technology in regions most affected by HIV, have led the World Health Organization to include SMS text message reminders as a recommendation for promoting adherence to ART as part of a package of adherence interventions [11].

There is limited understanding of the message content desired by text message recipients, or the mechanism by which messages may impact ART adherence, especially in the context of pregnancy and the postpartum period. While the design of text messaging interventions for ART adherence for PMTCT may draw on lessons learned from studies in other HIV-infected populations, the unique context of pregnancy and postpartum may influence ART adherence, and text messaging may need to be adapted accordingly. Few previous studies have explored the desired text message content to support women’s uptake of PMTCT services [12-15]. These studies reported a desire for polite, encouraging SMS text messages that provide information and reminders to take ART, attend postnatal visits, and engage partners. While health care workers (HCWs) and male partners are have been shown to play important roles in influencing peripartum women’s health behaviors and health care utilization [16-19], only two studies, to date, have included these stakeholder groups in evaluating the acceptability and desired content of text messages [12,15].

In this study, we examined the preferred SMS text message content and perceived text message function to support Option B+ PMTCT. We incorporated the perspectives of HIV-infected pregnant and postpartum women, male partners and HCWs.

## Methods

### Study Design and Population

We conducted a qualitative study to inform the text message content for the Mobile WACh-X study (NCT02400671), a triple-arm, placebo-controlled, unblinded RCT designed to assess the impact of unidirectional and bidirectional SMS text messaging on maternal adherence, retention, and clinical outcomes in PMTCT-ART programs in Kenya [20]. Using purposive sampling, we recruited women, male partners, and HCWs from three sites; two in rural Western Kenya and one in periurban Nairobi. Focus group discussions (FGDs) were conducted with HIV-infected pregnant women seeking antenatal care (ANC) services or HIV-infected postpartum women who had an uninfected child aged ≤2 years. Women were purposively recruited during routine visits to ANC clinics, comprehensive HIV care clinics, and maternal child health (MCH) clinics. To provide a range of experiences and perspectives, we selected pregnant and postpartum women based on the following experiences with ART: using ART in the peripartum period only; using ART within and outside of the peripartum period; and no ART experience. Women were eligible to participate if they were aged ≥14 years, were HIV-infected and pregnant or postpartum, had daily access to a mobile phone, and were willing to receive SMS text messages.

We conducted semistructured individual interviews with male partners and HCWs. Both HIV-infected and -uninfected male partners were recruited for participation. We recruited HIV-infected men in concordant relationships during their routine HIV clinic visits. In addition, HIV-uninfected men were referred to the study by HIV-infected female partners attending MCH clinics; female partners were given a referral form inviting male partners to the clinic to learn more about the study. Eligible men were aged ≥18 years and had an HIV-infected female partner who was pregnant or had a child aged ≤2 years and was accessing ANC or MCH services. We purposively recruited providers aged ≥18 years from ANC and MCH clinics where they worked. Men were eligible to participate if they were directly involved in caring for HIV-infected pregnant women or HIV-exposed infants. Overall, 87 women participated in 10 FGDs (6-10 women per FGD); 15 men and 30 HCWs participated in semistructured individual interviews.

### Data Collection

We conducted two rounds of data collection between January and June 2015. In the first round, 6 FGDs were conducted with HIV-infected women, 15 individual interviews were conducted with male partners, and 30 individual interviews were conducted with HCWs. The objectives of the first round of data collection were to explore general opinions about health-related SMS text messages, determine comprehension and acceptability of predeveloped text messages, and elicit ideas for additional messaging themes in order to refine the message content. A second round of 4 FGDs elicited women’s feedback on the refined message content.

Both FGDs and interviews were conducted using a semistructured discussion guide including open-ended questions exploring three main topic areas: (1) challenges and resources for attending a clinic and adhering to ART, (2) perspectives on using SMS text messaging to support adherence, and (3) perceptions of specific message content to guide message refinement. We asked participants to provide feedback on messages in four content areas: general support, breastfeeding, family planning, and ART adherence. All messages shared a common format: they opened with a greeting to the recipient from a nurse (“[Name], this is [nurse name] at [clinic name]”), followed by a message addressing one of the content areas (Supplementary Material 1) [20]. Interviews and FGDs were conducted by a trained Kenyan social scientist who was not involved in providing clinical care for participants. Pilot messages were read aloud by the discussion facilitator, and participants were probed for additional message content they would like to receive, beyond what was included in initial pilot messages. Sociodemographic information for all participants was collected via a tablet-based questionnaire using Open Data Kit. Interviews and FGDs were conducted in English, Kiswahili, and Dholuo, depending on participants’ preference. FGDs ranged from 90 to 130 minutes in length, and interviews ranged from 19 to 49 minutes. All interviews and FGDs were audiorecorded, transcribed, and translated into English, if necessary, by the interviewer, who was fluent in all three languages.

### Ethical Considerations

This study was reviewed and approved by the University of Washington Institutional Review Board and Kenyatta National Hospital and University of Nairobi Ethics and Research Committee. All study participants provided written informed consent.

### Statistical Analysis

We performed a descriptive content analysis to identify key concepts emerging between and across groups of women, male partners, and HCWs. Dedoose software (version 7.6.6, Sociocultural Research Consultants LLC, Los Angeles, CA) was used for data management and analysis. An initial codebook was deductively and inductively generated by JF, KBS, and KR after reviewing the literature and reading a subset of FGD and interview transcripts. Next, the codebook was refined iteratively by reviewing additional transcripts and revising initial codes. We used the final codebook to perform consensus coding and facilitate discussion until reaching an agreement on the code application. All transcripts were coded independently by one team member (JF, KBS, or KR) and reviewed by another team member. All disagreements in code application were resolved through group discussion with all three coders. The analytic framework focused on challenges living with HIV, current resources or strategies used to engage in HIV care, preferences and perceived utility of the specific SMS text message content, and benefits or challenges to using SMS text messaging to engage in care. Furthermore, we identified themes related to the analytic framework categories and combined them into a conceptual diagram.

## Results

### Participant Characteristics

Demographic characteristics of women, male partners, and HCWs are summarized in Table 1. Female participants (N=87) were young (median age, 26 years), most (64/87, 74%) had completed at least primary education, and about one-third (30/87, 34.5%) were pregnant. The majority (60/87, 69%) had experience with ART during and outside of the peripartum period, and a little over half (48/87, 55%) were Dholuo speakers. Male participants (N=15) were older than female participants (median age, 37 years) and had similar levels of education (12/15, 80%, had completed at least primary education); most (12/15, 80%) were HIV-infected and had experience with ART. The median age of HCWs was 36 years, and they had a median experience of 6 years in their current profession. Providers included clinical officers (n=7), nurses (n=13), counselors (n=6), a peer counselor (n=1), and others (n=3).

### SMS Text Messaging is Acceptable but Design Requirements and Challenges Exist

Overall, participants in all stakeholder groups (women, male partners, and HCWs) were supportive of using SMS text messages to overcome HIV-related challenges. Women generally found SMS text messaging acceptable and discussed its many potential benefits, including enabling remote connection to HCWs and providing anonymity that facilitates open communication about sensitive or potentially embarrassing topics.

It is eas[ier] to share information about shameful diseases by SMS than telling the doctor face-to-face. For instance, when I have a wound in my private parts…I can send him a message and he gives me a response immediately to use a particular medicine.

Postpartum woman

[The SMS messages] keep records of treatment and conversations for future reference for the patients, and also helps you to identify your patients individually and that creates a bond between you and your patients; they feel that you have them at heart, and then they feel that you are concerned about their health.

Clinical officer, 4 years in profession

**Table 1:** Focus group and interview participant characteristics.

Participant characteristics			Value
**Female FGD^a^ participants (N=87)**			
	Age (years), median (IQR^b^)		26 (23-32)
	Pregnant (vs postpartum), n (%)		30 (35)
	**ART^c^ experience, n (%)**		
		Peripartum only	19 (22)
		Peripartum and nonperipartum	60 (69)
		None	8 (9)
	**Education, n (%)**		
		Less than primary	23 (26)
		Primary completed	43 (49)
		Secondary completed	18 (21)
		Above secondary	3 (3)
	**Language, n (%)**		
		Dholuo	48 (55)
		Kiswahili	39 (45)
**Male interview participants (N=15)**			
	Age (years), median (IQR)		37 (32-44)
	**Education, n (%)**		
		Less than primary	3 (20)
		Primary completed	8 (53)
		Secondary completed	2 (13)
		Above secondary	2 (13)
	HIV-infected, n (%)		12 (80)
	ART experienced, n (%)		12 (80)
**Provider interview participants (N=30)**			
	Age (years), median (IQR)		35.5 (31.0-44.0)
	Years in profession, median (IQR)		6 (4.0-15.5)
	**Profession, n (%)**		
		Clinical officer	7 (23)
		MCH^d^ nurse	6 (20)
		Other nurse	5 (17)
		Family planning nurse	2 (7)
		Counselor	6 (20)
		Peer counselor	1 (3)
		Other	3 (10)

^a^FGD: focus group discussion.

^b^IQR: interquartile range.

^c^ART: antiretroviral therapy.

^d^MCH: maternal child health.

**Figure 1:**
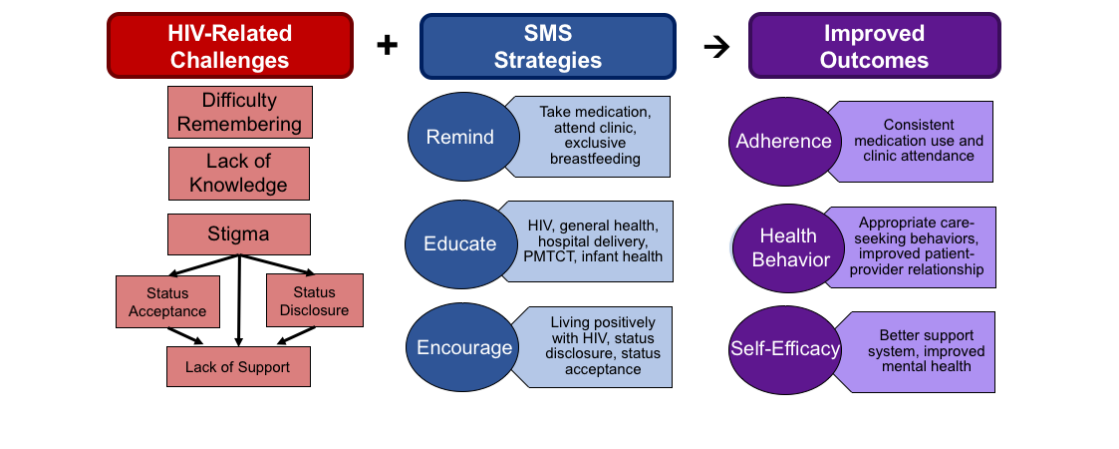
Summary of themes discussed in focus group discussions and interviews. PMTCT: prevention of mother-to-child HIV transmission. SMS: short message service.

However, a few providers expressed concerns about the ability to reach patients using SMS text messaging, specifically noting inability to confirm message receipt and challenges related to requirements for patients to have a phone, be literate, and have access to electricity.

I don’t know whether the SMS can work, but maybe a phone call [is better] so that you are very sure that the message has reached the client…comparing the two, I prefer the phone call. However, automated messages has worked in several occasions, in other fields, but what I am saying that it depends on the people or rather the region you are dealing with. If they have phone and [are] staying in a powered house, it is well and good, but in villages that may be a setback.

Nurse, 8 years in profession

While many participants were supportive of delivering health education on HIV care, several women and HCWs expressed concerns about SMS text message privacy and risks of HIV status disclosure if messages were read by people other than the intended recipient. All 3 stakeholder groups commented that the risk of inadvertent disclosure by other individuals reading SMS text messages should be minimized and the phrasing of HIV-related health education should be tailored based on the recipients’ disclosure status and desires. These concerns are discussed in detail in another publication [21].

Figure 1 summarizes topic areas included in the interview guides and related themes that emerged during FGDs and interviews. The figure shows the challenges that participants identified for women receiving PMTCT care, potential roles for text messages to address these challenges, and possible pathways through which text messaging may impact clinical outcomes. Discussions on the desired text message content highlighted three types of messages with different perceived functions: (1) reminders for medication and clinic attendance; (2) health education messages; and (3) encouragement to remain in care, seek support, and live positively.

### SMS Text Messages May Serve as Adherence Reminders During the Stressful Peripartum Period and Avoid Stigmatizing HCW Interactions Due to Missed Visits

Women identified forgetting to take medication or attend clinic as a major challenge in ART adherence and believed that SMS text message reminders could help overcome this obstacle. They attributed forgetfulness to various stressors, including some stressors unique to or heightened during pregnancy or postpartum, such as caring for newborns.

There are…those who take…medicine, but considering how busy they are, she is the breadwinner, so sometimes she is occupied until time passes. She forgets, so when you send her a message she will remember “tomorrow I am to go to the hospital.” Like here…some of us go to work on the rice farms, others to the farm, others casual labor, maybe that is what she depends on because she is the mother and the father. So when she receives a message, it will alert her.

Postpartum woman

Women also described negative interactions with HCWs, including feeling stigmatized and being treated harshly, if they missed appointments or delayed seeking HIV care. Women and providers suggested that text message reminders to attend scheduled appointments could help prevent conflict with providers.

I think that SMS is better than the card that we are given because sometimes you leave out the card somewhere and you forget and by the time you remember it has passed by one day, and if you are reminded you will be quarreled [with] at the hospital, yet it is just forgetfulness that makes you to default.

Postpartum woman

Providers concurred with the sentiments expressed by women, noting that text messages could reduce missed visits due to forgetfulness and ensure timely follow-up for women.

I think for SMS…it’s good for them because some they tend to forget, others…they say I didn’t know when to attend the clinic, [it’s] when I have seen my bottle is empty, or I have seen my child…is 10 months already (you know at 9 months that is the time when you have to bring the child for [HIV] antibody [testing])…So when the SMS is there, the services will be at the right time and evaluation will be done early.

Nurse, 4 years in profession

Both women and their partners noted that male partners had an important role in reminding women to take medication and attend clinic if women disclosed their status to them. Indeed, male partners thought that text messages could complement the role they played in reminding their partners, especially when they could not be physically present.

Just like sometimes someone may forget, so you send a message to alert her that on a particular day she should be going to the clinic. So that would help her, it would also help me because I may not be at home to remind her that she should go for medicine or to the clinic.

HIV-infected male partner

### SMS Text Messaging May Provide Health Education About PMTCT-ART, Information on Clinical Status, Answers to Questions, and May Catalyze Discussions With Others

Women expressed a desire for educational messages to fill gaps in their knowledge and understanding about HIV care, including obtaining information about common ART side effects, modes of MTCT, and effective PMTCT strategies. Women believed that educational SMS text messages could inform them about PMTCT and the ability to have healthy, HIV-uninfected babies even if they are HIV-infected and that improved knowledge would increase motivation to engage in care throughout pregnancy and breastfeeding.

What I would like to know, I thought that if you are sick then you cannot give birth to a healthy baby, but I hear that if you are on HIV care and treatment, then you can give birth to a healthy baby who is HIV negative.

Postpartum woman

Women frequently stated that they wanted to receive SMS text message advice on how to discuss HIV and ART with partners. Some women believed that SMS text messages containing credible information could be shared with their partners and served as a catalyst to engage in conversation.

I can say that [SMS] can help. My husband has refused to accept [being tested], but he helps me financially when I have hospital appointments. So I think that the SMS can help: if I give him to read he can be encouraged and he may decide to come out and know his status.

Postpartum woman

Additionally, several participants viewed SMS text messages as a platform to receive updates on their viral load, CD4 count, and treatment status. Delivery of test results through SMS text messages was seen as a way to remove physical barriers to the clinic and prevent delay in information that women could use to make personal health choices in real time.

Sometimes I go to do CD4 test, and I will get the result later…so I can ask by SMS before my clinic date and get the results, so when the time for clinic reaches if the doctors asks me if I know my CD4 results I will say that it [is] like this and this.

Postpartum woman

Both male partners and HCWs found it acceptable and beneficial to deliver educational content to women via SMS and highlighted the importance of delivering this information quickly and efficiently since traveling to a clinic for basic health information was often burdensome. Some participants suggested that using SMS text messages to address minor health questions could enable triaging and avert unnecessary clinic visits.

[SMS] is good, it is one-on-one. If you have any problems [the provider] can help you, you can ask questions, and you just handle it then when you are at home without going to the hospital. It might be small issues like rashes you handle and need not go to the hospital.

HIV-uninfected male partner

Similarly, HCWs commented that delivering educational information via SMS text messages would be beneficial not only to women but also to themselves, as it would ease their workload.

To me, I think…use of SMS, it will be good. It will be good [be]cause sometimes…mothers come to flock here, [be]cause of even minor illnesses that you could have just given advice…So in order to decongest MCH, such minor cases maybe if we can use SMS, it will be of a great [help] to us…

Nurse, 20 years in profession

### SMS Text Messages May Provide General Encouragement to Improve Self-Acceptance and Combat Stigma, Which, in Turn, Could Motivate ART Adherence

Many women thought that text messages could provide encouragement and motivation to help engage in HIV care, adhere to ART, and accept their status. Although women reported that encouraging text messaging would help them engage in HIV care, they did not feel this required language directly addressing HIV-related topics. Rather, many women expressed desire for text messages providing encouragement to live a healthy and positive life while pregnant or postpartum, to stay strong and have a positive outlook on life for themselves and their children, and to not lose hope. Women frequently mentioned that they would feel happy and encouraged if they received an SMS text message from their provider, even if it was simply to ask about potential challenges they may be facing (related or unrelated to their HIV care). In addition, many women commented on the potential of text messages to provide a sense of connection and acceptance from the sender during times of hopelessness or isolation, highlighting that this connection would lift their morale and improve their ability to engage in HIV care.

If you receive the message…you will not feel lonely, you will know that there is someone who is concerned about you and that despite being HIV positive you are not alone.

Postpartum woman

Indeed, some participants indicated that their sense of feeling cared for grew out of the knowledge that the sender had invested time to send a text message, rather than the use of encouraging language *per se* in the message.

[SMS] will help to encourage me that someone was concerned about me by taking their time to send the messages. It costs you, and I get help. So, I think that it helps to encourage knowing that someone somewhere is concerned and has accepted me.

Pregnant woman

Similarly, some male partners indicated that text messages could encourage and support women struggling with status acceptance and help them identify other sources of support.

The messages…should be sent to let [people] know that they are not alone. So many people are undergoing what they are going through so they are not left alone, and there are people who are always there to assist them…[S]o they should accept the situation and try as much as they can to maybe look for people who…can be there for them.

HIV-infected male partner

HCWs placed a stronger emphasis on using text messages to provide reminders and health education than on their use for emotional support. However, some providers noted that text messages had the potential to serve as an encouragement system, working beyond “tracing defaulters.”

I think [SMS] will be really good for the newly diagnosed mothers to give them that psychosocial support that they lack. Because we usually see them after 2 weeks—you know [in] 2 weeks there is so much that can happen—so that by the time she is coming back after 2 weeks, she is really happy.

Nurse, 25 years in profession

### General MCH SMS Text Message Content Was Felt to Be Useful in Addition to PMTCT Content

Although discussions with all participants were framed as addressing experiences of HIV-infected women with PMTCT, women, men, and HCW repeatedly expressed a desire for text messages to support general MCH care in addition to supporting HIV care. The topics requested included breastfeeding instructions, challenges to exclusive breastfeeding, and maternal nutrition. Functionally, the MCH-related messages that participants suggested were mostly educational rather than reminders or supportive messages.

[Please include messages] about nutrition during pregnancy: what a woman can eat so that at the time of birth she has enough strength to deliver and enough milk to breastfeed the baby.

Pregnant woman

How can we help a baby who is not feeding? Because you cannot tell it to breastfeed, yet when you give it breastmilk it does not suckle, yet this first milk is what it must take, so the doctor should help us with that because we don’t know.

HIV-infected male partner

## Discussion

### Principal Results

In this study, HIV-infected women, male partners, and HCWs in Kenya endorsed SMS text messaging as a way to optimize retention and adherence to PMTCT-ART. Participants proposed the following three text message content areas that could serve complementary functions: reminders, education, and encouragement. In addition, all participants endorsed text messages as a useful mechanism for reminding women to take medication and return for clinic visits. Text message reminders were reported to be particularly useful in the peripartum period, when the stresses of delivery and newborn care increase forgetfulness. Additionally, women and HCWs indicated that difficult interactions following missed visits could be avoided with reminder text messages. Health education was also valued, including reinforcement of the importance of ART to prevent infant HIV and the likelihood of success in having a healthy baby. Women desired timely access to their clinical data and to interactive messaging to ask questions without the need for an in-person clinic visit or embarrassing face-to-face conversation. Additionally, text messaging was viewed as a promising intervention to engage and inform partners, if disclosed, to leverage their influence on maternal ART adherence [18,19,22]. Text messaging was also viewed as an important enabler of emotional support and encouragement. Women perceived text messages as providing a connection with HCWs and validating that they were worthy of care, which improved their mental health, helped them overcome internalized stigma, and increased their motivation to adhere to treatment. Importantly, in addition to HIV-related topics, women, partners, and HCWs were interested in SMS text messages that supported women’s general MCH care, for example, breastfeeding, maternal nutrition, and facility delivery.

### Comparison With Prior Work

Previous qualitative studies have explored the acceptability and content preferences of SMS text messaging interventions to improve ART adherence in the general HIV-infected population, reporting desired content prior to text messaging interventions [23,24], or postintervention perspectives [25]. Four studies focused on text message content preferences to support PMTCT [12-15]. Of these, two were conducted in Kenya prior to roll-out of Option B+ PMTCT, when PMTCT involved short-term antiretroviral regimens during the period of highest risk of MTCT [12,13]. One study interviewed postpartum women, male partners, and HCWs and reported that all were supportive of SMS text messages as appointment reminders and as education providers regarding ANC, facility delivery, and partner engagement [12]. The other study only involved peripartum women and reported that they desired encouraging, educational messages about postpartum visit attendance and infant HIV testing [13]. Our findings were similar to those of these studies in that participants desired text messages regarding general MCH topics as well as HIV-related information. Additionally, we found that women desired text messages for emotional support in the face of isolation and internalized stigma. Two studies were conducted after Option B+ roll-out: one interviewed pregnant women in South Africa [14] and the other interviewed women, partners, and HCWs in Kenya [15]. These studies reported a desire for text messages as reminders, information, and encouragement, but did not identify a need for messaging about general MCH topics beyond HIV care. Our findings are consistent with those of these studies as well and provide additional insights regarding the need for encouragement and messaging related to MCH.

One potential implication of the differences between our findings and those of previous studies may be that the need and perceived utility of text messages for emotional support are greater in the context of long-term ART adherence than in short-course PMTCT. Indeed, the ability of SMS text messages to serve a supportive, caring function regardless of the message content has been suggested by previous studies on SMS text messaging for long-term adherence support in the general HIV-infected population. A qualitative evaluation of the experiences of recipients of real-time adherence monitoring and SMS text message reminders showed that recipients interpreted simple reminder messages as a sign of caring from the health system [25]. Similarly, results from the WelTel trial, one of the first studies to report that an SMS text messaging intervention improved ART adherence, suggested that participants viewed a sense of support and caring as an important function of text messages [26-28]. These findings are consistent with the association of depression and low social support with poor ART adherence reported in some studies [29-31].

PMTCT is HIV care in the context of pregnancy, childbirth, and postpartum. Many women receiving PMTCT perceive these life events as at least as important, if not more important, than their HIV infection. Thus, addressing HIV without acknowledging concerns about pregnancy, delivery, and infant health in text messages does not address women’s needs for holistic care. In this study, women desired text messages on MCH topics such as breastfeeding, infant health, and maternal nutrition. It is perhaps unsurprising that women’s experiences and needs as peripartum women were not fully defined by their HIV status, but this observation highlights the demand and untapped potential for SMS-based interventions to provide comprehensive care to peripartum women. An important question for future investigation is whether in addressing both MCH and ART adherence, SMS text messages lose focus and effectiveness to improve ART adherence.

### Strengths and Limitations

This study has several strengths and limitations. The qualitative design allowed for in-depth understanding of SMS text message perceptions and content preferences among HIV-infected pregnant and postpartum women. We purposively sampled both pregnant and postpartum women with a range of ART experiences to capture a wide array of perspectives. Additionally, the inclusion of HCW and male partner perspectives provided complementary insights into how text messages may improve the delivery of Option B+ and fit into the broader context of influences on women receiving care. Study limitations include the possibility that participants felt uncomfortable discussing personal experiences if they perceived these as criticisms of the health care facility. This was mitigated by having interviewers who were unrelated to the facility and conducting interviews in a private room. Furthermore, the study population was drawn from women engaged in care and may not be generalizable to women out of care. Similarly, the recruitment of men by referral from their female partners likely excluded men who were unaware or unsupportive of their partners’ care; these men may have different views about their partners receiving SMS text messages. Finally, the desired text messages are not necessarily effective, and it will be important to further examine not only whether text messaging improves outcomes but also which text messages are specifically helpful. Nevertheless, inputs from women, partners, and HCWs are essential to design messages that address a felt need and have relevance.

### Conclusions

In summary, our findings support the use of SMS text messaging to enhance PMTCT and general MCH care. SMS text messages may meet women’s desires for medication and appointment reminders, health education, and emotional support and may complement partners’ supportive roles and enable efficiencies in HCWs’ care provision. Moreover, our findings shed light on the unique needs of HIV-infected peripartum women and indicate that these women desire support for not only HIV and PMTCT care but also their general health and their children’s health. In addressing these desires, text messaging approaches can provide comprehensive PMTCT and MCH support, but may face challenges in balancing and focusing effective messaging strategies to improve ART adherence.

## Appendix

Multimedia Appendix 1 Focus group and interview guides.

## Abbreviations

ANC: antenatal care
ART: antiretroviral therapy
FGD: focus group discussions
HCW: health care workers
MCH: maternal child health
MTCT: mother-to-child HIV transmission
PMTCT: prevention of mother-to-child HIV transmission
RCT: randomized controlled trials
SMS: short message service
