# Supplementary material for: You Will Know That Despite Being HIV Positive You Are Not Alone: Qualitative Study to Inform Content of a Text Messaging Intervention to Improve Prevention of Mother-to-Child HIV Transmission
Source: JMIR Mhealth Uhealth. 2018 Jul 19;6(7):e10671. doi: 10.2196/10671 (PMC6072973; doi:10.2196/10671)
Supplement: Multimedia Appendix 1 [file mhealth_v6i7e10671_app1.pdf]

Supplementary material 1: focus group discussion guides

FEMALE FGD GUIDE: Round 1

We are trying to explore ways in which mobile phones could be used to assist HIV + women to care for themselves and their babies, adhere to medications, and come to clinic. A lot of women may experience challenges in doing this, and we are wondering if SMS messages could be used to help reduce these challenges. We are interested in hearing your thoughts about challenges to HIV care and adhering to ARVs, and whether you think SMS could be useful, and how it could be used.

1) There are many ways that HIV+ women may experience challenges in taking care of their health. What do you think some of these might be?
    a. Coming for appointments
    b. Taking medications
    c. Emotional/psychological challenges
    d. Stigma/isolation
2) What are some of the reasons that women don't come in to clinic for their HIV care?
    a. What are some of the challenges when women are pregnant?
    b. What are some of the challenges after women have had their babies?
    c. What are some of the challenge women face when they are in general HIV care?
3) What challenges do women face with medication adherence?
    a. What are some reasons women do not take their medications?
        i. Probes
            1. Access
            2. Stigma/ non-disclosure
            3. Denial/ uninformed
            4. Depression
            5. Side effects
    b. What strategies do women use to overcome these challenges?
            1. Personal strategies –
            2. System strategies – (M2M, counseling)
            3. Other novel strategies-
4) How can SMS be used to support women's adherence and clinic attendance?
    a. Probes
        i. Enhances provider and patient relationship
        ii. Women receive encouraging messages
        iii. Women receive advice
        iv. Visit reminders
        v. Women feel cared for
        vi. Others
5) If we were to send pregnant and postpartum women SMS messages, what kind of messages would help women adhere to medications and come to clinic?
    a. Probes
        i. HIV care related
            1. Side effects of medication
            2. I<u>ndirec</u>t medication and clinic appointment reminder
            3. <u>Direct</u> medication and clinic appointment reminder
            4. Prevention of transmission
            5. Acute health related issues
            6. Managing stigma
6) What other types of messages would women like to receive?
    a. Probes
        i. Pregnancy and infant related
            1. Danger signs in pregnancy
            2. Hospital or skilled delivery
            3. Nutrition advice and vaccinations
            4. Preventing HIV transmission and DNA PCR testing
            5. Infant illness
        ii. Other topics
7) How do you think women would feel about receiving messages that explain HIV or their specific HIV medications? What concerns would they have?
    a. Probes
        i. Better / more detailed information

    ii. Risks of confidentiality

8) Would women be willing to ask specific questions about HIV over SMS? Why or why not?
9) What are the potential benefits for women to be able to communicate with nurses via SMS?
    a. Probe: Medications and visits
        i. Reschedule missed appointments
        ii. Report adverse outcomes
        iii. Support for medication non-adherence
    b. Probe: Sensitive topics
        i. Women can use SMS to communicate with providers
        ii. Some women might find it easier to seek advice on sensitive topics via SMS rather than in-person
        i. Probe: Urgency of required information: STI management, breastfeeding questions, danger signs in pregnancy, intimate partner violence
    c. Probe: access
        a. get a more rapid response than going to the clinic
        b. avoid travel cost and time of going to clinic
        c. avoid unpleasant interactions with staff at clinic
10) What are some possible barriers to use of SMS?
    a. Probes
        i. Inability to read and write SMS
        ii. Loss of confidentiality and stigma
        iii. Loss of phone
        iv. Phone lacking charge/ power outage
        v. Provider-patient conflict
11) What do you think your partner would do if got he got the SMS instead?
12) Would you want to share the messages with your partner?
13) All messages will be addressed to you. Would you also want your partner's names to be included in the messages?
14) Would you be willing to share SMS health information with other people?
    a. Who?
    b. Which pieces of information would you share?

Next, we will show you some predesigned SMS texts that focus on your health and care.
NOTE: For each message, ask the following:

15) What does this mean?
    a. Was it easily understandable?
    b. Which parts were confusing?
    c. What message is this trying to convey?
    d. What behavior is this message trying to impact?
16) How best could this be phrased?
17) What do you feel about the length of message?
18) Do you feel the messages are personalized?
19) What other topics should be added?

<u>General questions</u>

20) What can be done to make SMS more effective?
21) Given a choice, what would you prefer? Voice calls or SMS?
    a. Why?
22) Do you have any concluding remarks or questions regarding our discussion today?

Conclusion
Thank you very much for your time and for all the helpful information you have provided. We will use this information you have provided in combination with information from others, to recommend solutions for better care of women and their children.

Male IDI guide: Round 1

1) What strategies have been useful in helping you adhere to your care? (If HIV+)
2) If we were to send SMS to you partner what types of messages could help your partner adhere to her treatment and clinic appointments?
3) Have you tried any strategies to help your partner adhere to her treatment and appointments?
4) Would you utilize SMS to communicate with your health care provider if it was available to you?
5) If your partner was to participate in this type of SMS study, what concerns would you have?
6) Would you also want to receive SMS message for your HIV care?
7) How would you feel about receiving SMS that mentioned HIV?
8) How do you think your partner would feel about receiving these messages?
9) Would it be helpful if your partner shared the messages with you? Why?
10) What are your thoughts regarding the sample messages we have shown you?

Provider IDI guide: Round 1

1) We would like to know what strategy you are using for PMTCT and HIV care for pregnant and postpartum women? Is PMTCT Option B+ the standard of care in your facility?
    a. If yes, how is it going? What are the challenges you are having? (Probes: With counseling? Medication? Adherence? Loss to follow up?)
    b. Where do women get HIV care when they are pregnant? Postpartum?
    c. What happens after the postpartum period?
2) Once a woman is diagnosed with HIV what counseling process does she undergo?
    a. What support do you provide?
3) What is the current workload with respect to HIV/ ARV counseling for HIV infected women initiating PMTCT/ ARV?
4) On average how much time do you spend counseling the woman about PMTCT/ARV or HIV?
5) Do you think you have sufficient time to counsel the mother prior to initiating ARVs?
6) What counseling messages are you currently providing to HIV-infected women?
    a. Probes
        i. HIV related
            1. Importance of medication adherence and clinic visits
            2. Prevention of HIV transmission
            3. Infant PCR testing
            4. Disclosure
            5. Managing stigma (perceived or experienced)
        ii. contraception
7) Which additional HIV related counseling messages would you deliver if you had additional time?
8) Do you think SMS could be used to deliver these additional messages?
9) What types of messages would you think would be helpful based on what you see at the clinic or what women go through?
    a. Probes:
        i. Encouragement messages
        ii. Educational messages
        iii. Visit reminders
        iv. Medication management
10) Would you like to be able to communicate with patients via SMS? Are you currently communicating in this way?
11) Do you ever explicitly SMS about HIV? Would you be willing to do this? Do you think patients would like to SMS about their HIV disease, challenges with HIV and medications?

Next, we will show you some predesigned SMS texts. Note, the words, HIV, ART or any term related to HIV will not be mentioned.

For each message please explain:

12) What does this message mean?
13) What type of behavior do you think this message targets to change?
14) What other types of messages would you want to see or add?
15) What are your concerns regarding use of SMS?
    a. Probes
        i. Inadvertent disclosure of HIV and other sensitive information
        ii. Loss of confidentiality and stigma
        iii. Provider work load or burn out with two-way SMS
16) What are potential barriers to use of SMS?
    a. Probes
        i. Loss of phone
        ii. Phone lacking charge/ power outage?
17) Is there any other way this type of message or system could be helpful to you as a provider?
18) Do you have any concluding remarks or questions regarding our discussion today?

Conclusion

Thank you very much for your time and for all the helpful information you have provided. We will use this information you have provided in combination with information from others, to recommend solutions for better care of women and their children.

Round 1 SMS messages

1. <Name>, this is <nurse> from <x> clinic. Take time each day for your health. If you are having challenges, let us know.
2. <Name>, this is <nurse> from <x> clinic. If you are having any health concerns we are available to help.
3. <Name>, it is <nurse> from <x> clinic. You are to come in XX day XX date. We will give vaccines to your baby. You may also get free family planning. Please come in.
4. <Name>, it is <nurse> from <x> clinic. We missed you in clinic today. You are due for your visit. Please come in so we can check you and your baby.
5. <Name>, this is <nurse> from <x> clinic. Breastfeeding a baby right after birth helps the milk come. The first yellow sticky milk has many vitamins & cleans out the stomach. Milk has all the nutrients the baby needs.
6. <Name>, this is <nurse> from <x> clinic. It's time for your baby's clinic visit in 2 days. Your baby will receive a vaccine and check growth. If you have questions about your baby's health, ask the nurse.
7. <Name>, this is <nurse> from <x> clinic. The IUCD or coil is a small device for family planning. Easy to put in, safe and very effective for years but can be removed at any time! Ask your nurse.
8. <Name>, this is <nurse> from <x> clinic. Regular, strong contractions are a sign of labour. If you feel strong tightening of your belly, leaking of fluid or any bleeding go to the clinic. Don't wait.
9. <Name>, this is <nurse> from <x> clinic. Take time each day to take your medication. It will keep you healthy If you are having side effects, let us know.
10. <Name>, this is <nurse> from <x> clinic. If you are having any helth concerns about HIV or your medications we are available to help.
11. <Name>, this is <nurse> from <x> clinic. Take time each day for your health. If you are having challenges, let us know. Have you had any challenges this week?
12. <Name>, this is <nurse> from <x> clinic. If you are having any health concerns we are available to help. Are you feeling well this week?
13. <Name>, it is <nurse> from <x> clinic. You are to come in XX day XX date. We will give vaccines to your baby. You may also get free family planning. Please come in. Are you coming for your visit?
14. <Name>, it is <nurse> from <x> clinic. We missed you in clinic today. You are due for your visit. Please come in so we can check you and your baby. Are you having difficulty coming to clinic?
15. <Name>, this is <nurse>from <x> clinic. Iron helps carry nutrition to your baby. If it is low, you feel tired. Are you taking iron or do you need tablets?
16. <Name>, this is <nurse> from <x>clinic. If your new baby is having trouble breathing or feeding, is too warm, or very sleepy, go to the clinic. Does the baby have trouble feeding? How many times a day does the baby feed?
17. <Name>, this is <nurse>from <x> clinic. The Implant is a small rod with medicine for family planning. It is placed in your arm and is very effective for 3 years. The Implant can be removed at any time! Would you like to try the Implant? Do you have any questions about it?
18. <Name>, this is <nurse>from <x> clinic. Delivery at the clinic or hospital could save your baby’s life. Do you have any questions about where to go in labor and how to get there?
19. <Name>, this is <nurse> from <x> clinic. Take time each day to take your medication. It will keep you healthy . Are you having any side effects?
20. <Name>, this is <nurse> from <x> clinic. If you are having any health concerns about HIV or your medications. Are you having any probelms?

FGD GUIDE: Round 2

We are developing a study to assist HIV+ women to care for themselves and their babies, adhere to medications, and come to clinic. Our plan is to offer HIV+ women information and support by SMS messages sent to their phones. In this focus group, we are asking for your opinions in developing the messages that will be most helpful for these women.

In our previous focus groups, women told us that they would find it helpful to get messages about:

- pregnancy
- delivery
- feeding their babies
- preventing mother-to-child transmission of HIV
- reminders and encouragement to help women take their ART
- appointment reminders
- support with ART side-effects

1) Are there any additional topics that you think HIV+ women would like to receive messages about?

2) Now I'm going to show you some SMS messages that we have drafted, and we want to get your opinion of them [start with non-HIV messages, ask women directly "how can we remind women to adhere to medication without talking about hiv?"]

Probes

a. What do you understand from this message?
b. How could it be clearer or better?
c. How else might we remind women to take their medications or ask about side effects?

3) For family planning messages: we plan to have messages over a few weeks that discuss different methods each week. [show multiple messages to give women sense of the continuity]
    a. How do you think women's partners will react to these messages?
        i. If concerns – what concerns would they have?

4) Many women have said that messages about taking ART, ART side effects or PMTCT would be helpful. But some women we've talked to were concerned about messages that contain HIV-related words, in case someone sees the message and finds out the woman is positive. Other women said if women have disclosed to their partners, it's ok to get messages that mention HIV.
We are planning to give each woman the choice, as long as she has disclosed to her partner, whether to receive messages that talk openly about HIV, or messages that are more discreet. We will allow women to change their choice at any time in the study.
    a. What do you think are some benefits of HIV-related messages? What are some risks?
    b. What do you think about our strategy of giving women this choice?
    c. How many of you would choose to get HIV-related messages and how many wouldn't want it?

5) For those women who don't want HIV-related messages, which words do you think should be **avoided** so as not to arouse suspicion?
    Probes:
        i. 'medication'
        ii. 'dawa'
        iii. 'vitamin'

6) HIV-specific messages. **[**show HIV-related messages and get input on how to modify each]

- medication reminder/encouragement
- side effects
- prevention of mother to child transmission

For each of these messages

a. What do you think this message means? Would you find this message helpful? How could it be improved?
b. If we wanted to convey this information without saying HIV-related words, how do you think we could phrase it?
    i. For medication reminder, what about: "Take time each day for your health"?
    Probes:
        1. When you see this phrase, what do you understand from it?
        2. Does it make you think about taking ART each day?
        3. Is it clear enough?
        4. Is it discreet enough?

5. What would be alternative phrases?

7) There are some messages that are addressed both to the woman and her partner. What do you think of these? Do you think that women would want to have the message addressed to both her and her partner?

8) Do you have any concluding remarks or questions regarding our discussion today?

Conclusion

Thank you very much for your time and for all the helpful information you have provided. We will use this information you have provided in combination with information from others, to recommend solutions for better care of women and their children.

Round 2 SMS messages

1. Hi <Name>. This is <nurse> the nurse from <x> clinic. Your health is very important. Take time each day for your health. If you are having any challenges please let us know.
   Hi <Name>. This is <nurse> the nurse from <x> clinic. Your health is very important. Take dawa each day for your health. If you are having any challenges please let us know.
2. Good morning <Name> and <Partner Name>. This is <nurse> the nurse from <x> clinic. If you are having any challenges with your pregnancy or health, please talk to the nurse at clinic. We can help. Are you having any challenges now?
3. Hi <Name>. This is <nurse> the nurse from <x> clinic. Congratulations! Your baby is here! Your baby only needs breastmilk for the first six months. If you have any concerns about breastfeeding or want to give the baby something else, please come into clinic and talk to a nurse.
4. Hi <Name>. This is <nurse> the nurse from <x> clinic. Congratulations! Your baby is here! Your baby only needs breast milk for the first six months. How often is your baby feeding? Are you having any challenges to breast-feed?
5. Hi <Name>. This is <nurse> the nurse from <x> clinic. Over the next few weeks we will send you messages to help you make family planning decisions. Planning your family and spacing pregnancy is good for your health.
6. Hi <Name>. This is <nurse> the nurse from <x> clinic. The IUCD or coil is a small device for family planning. Easy to put in, safe and very effective for years but can be removed at any time! Ask your nurse. Do you know any one who has used the coil? Do you have any questions?
7. Hi <Name>. This is <nurse> the nurse from <x> clinic. The Implant is a small rod with medicine for family planning. It is placed in your arm and is very effective for 3 years. The implant can be removed at any time. Would you like to try the implant? Do you have any questions?
8. Hi <Name>. This is <nurse> the nurse from <x> clinic. Remember that ARVs keep you healthy and prevent your baby from being infected. We are here to help. Are you having any challenges taking your HIV medicines?
9. Good morning <Partner Name> and <Name>. This is <nurse> the nurse from <x> clinic. ARVs are the best way to keep you health and prevent your baby from being infected. We are here to help. Are you having any challenges with your medicines?
10. Hi <Name>. This is <nurse> the nurse from <x> clinic. If you feel sick during pregnancy, try and nibble on dry biscuits to help you take your medicine. Don’t stop taking your ARVs when you feel sick. Are you having any vomiting or other challenges?
11. Good morning <Name>. This is <nurse> from <x> clinic. Remember to take your HIV medicines every day to help you stay well for your baby and your family. We are here for you. Are you having challenges with your drugs?
12. Good evening <Name>. This is <nurse>, the nurse from <x> clinic. Your medications help your baby from becoming infected and do not harm the baby. Let us know if you have any concerns about your medicines.
